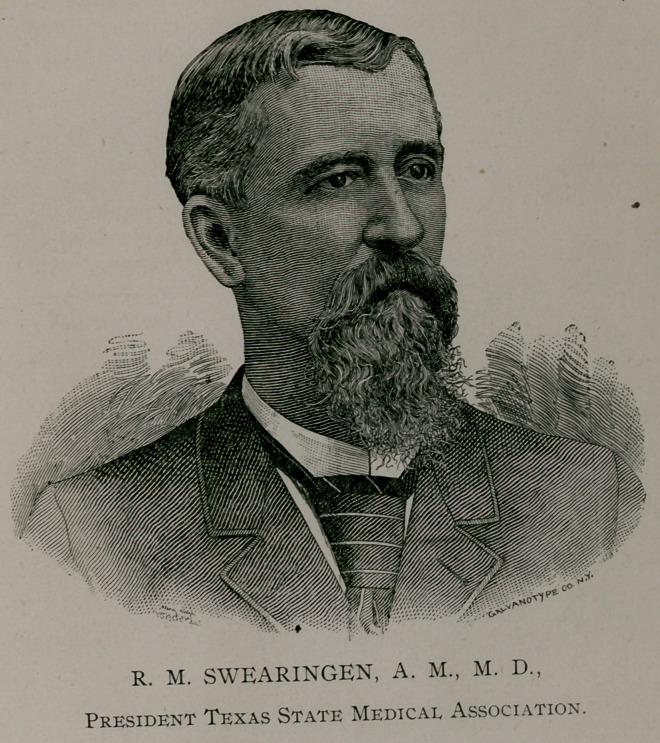# R. M. Swearingen, M. D., President Texas State Medical Association

**Published:** 1889-05

**Authors:** F. E. Daniel


PRESIDENT TEXAS STATE MEDICAL ASSOCIATION.

### RICHARD M. SWEARINGEN, A. M., M. D.

[Subject of Portrait.]

PRESIDENT TEXAS STATE MEDICAL ASSOCIATION.

[From the forthcoming work: Biography of Contemporary Physicians of Texas; By F. E. Daniel, M. D.]

The subject of this sketch was born in Noxubee County, Miss., Sept. 26, 1838. His parents emigrated to Texas in 1848. Dr. Swearingen studied medicine and attended a course ot lectures at the New Orleans School of Medicine in 1859-60, during the services of the Flints—Sr. and Jr.,—E. D. Fenner and other distinguished teachers. The war between the States coming on interrupted his studies, and he entered the Southern army as a private soldier, responding to the first call for volunteer troops in Texas, February 28, 1861, and, in the course of a few months, was promoted to the command of his company, receiving from the

War Department his commission as Captain of Cavalry. He remained until the close of the war in command of this, one of the finest cavalry commands in the service, and participating actively in the numerous campaigns in Tennessee, Kentucky, and Virginia; surrendered finally with Gen. Joseph E. Johnston, at Charlotte, N. C., when resistance was no longer possible.

During the war he married Miss Jennie Jessee, the daughter of a Tennessee gentleman, at whose house the Doctor was left sick on one occasion.

Upon the cessation of hostilities he returned to Texas, and locating in Washington county, in the town of Chapel Hill, he resumed the study of medicine with Dr. Rogers, and, attending a second course of lectures in 1867 at the New Orleans School of Medicine, was graduated M. D., with first honors, delivering the valedictory of his class.

Engaging immediately in the practice of his profession at Chapel Hill, Dr. Swearingen commanded at once a large practice. In the spring of 1875 he removed to Austin, the capital of Texas, where he now resides, still engaged in a general prastice, large, lucrative, and growing. He has been successful financially, and owns an elegant home, where he resides with his family. He has an only child—now Mrs. E. B. Robinson, a beautiful and accomplished lady. The doctor enjoys the confidence and esteem of the entire community, and of an ever increasing troop of friends.

In the yellow-fever epidemic of 1878, Dr. Swearingen and Dr. T. D. Manning volunteered their services to the Howards, and proceeded to Memphis to aid the stricken people. On arrival they were assigned to Holly Springs, with instructions to take charge and establish a hospital. The gallant and accomplished Manning falling early a victim in the unequal struggle, the entire labor and responsibility of the hospital service fell upon his companion, the subject of this imperfact sketch. The history of those sad scenes will never be written, but the services of these two Texas volunteer physicians, one of whom so cheerfully laid down his young life in the cause of humanity, are indelibly engraved upon the hearts and memories of a grateful community.

In January, 1879, Dr. Swearingen was appointed, by the President of the United States, a member of the "Board of Experts upon Epidemic Diseases," and assisted in preparing a report to Congress that met the expectations of the people, and received the unqualified endorsement of the medical profession.

In February, 1881, he was appointed by Governor O. M. Roberts State Health Officer of Texas, and in 1883 he was reappointed by Governor Ireland. For six years the control and administration of the extensive quarantine system of the State, extending over an immense line of sea coast, as well as an extensive inter-State and Mexican frontier, have devolved upon him, and the conspicuous exemption of the Texas people from epidemic disease during that time, as well as his reappointment, testify to the distinguished ability with which his trusts have been discharged. In addition to his official duties as stated, Dr. Swearingen is constantly engaged in a general practice, yet finds time for social enjoyment, into which he enters with all the zest of an intense Southern nature, and also to contribute to the literature of his profession, he being an active member of the County, State, and National Medical Associations; and was recently appointed by the Executive Committee of the International Medical Congress to an inportant position on the Section of State Medicine and Public Hygiene, a subject to which he has given much attention. He is regarded, at least throughout the South, as one of the foremost and most enlightened sanitarians of this progressive age.

Among his literary productions, his eulogy on the life and services of Dr. T. D. Manning, delivered, as essayist for the occasion, before the Texas State Medical Association at Belton, in 1884, stands conspicuous as a model of eloquence and pathos. It will go on record as one of the most chaste and merited tributes ever paid to departed worth.

At the recent meeting of the Texas State Medical Association, (San Antonio, April 26, 1889,) he was unanimously elected President.

---

Dr. S. W. Gross, of Philadelphia, died April 16, of phneumonia, aged 53.

## Figures and Tables

**Figure f1:**